# Nutritional Supplement of Hatchery Eggshell Membrane Improves Poultry Performance and Provides Resistance against Endotoxin Stress

**DOI:** 10.1371/journal.pone.0159433

**Published:** 2016-07-27

**Authors:** S. K. Makkar, N. C. Rath, B. Packialakshmi, Z. Y. Zhou, G. R. Huff, A. M. Donoghue

**Affiliations:** 1 Poultry Production & Product Safety Research Unit, Agricultural Research Service, USDA, Fayetteville, Arkansas, United States of America; 2 Department of Poultry Science, University of Arkansas, Fayetteville, Arkansas, United States of America; 3 Department of Veterinary Medicine, Rongchang campus of Southwest University, Rongchang County, China; Universidade de São Paulo, BRAZIL

## Abstract

Eggshells are significant part of hatchery waste which consist of calcium carbonate crust, membranes, and proteins and peptides of embryonic origins along with other entrapped contaminants including microbes. We hypothesized that using this product as a nutritional additive in poultry diet may confer better immunity to the chickens in the paradigm of mammalian milk that enhances immunity. Therefore, we investigated the effect of hatchery eggshell membranes (HESM) as a short term feed supplement on growth performance and immunity of chickens under bacterial lipopolysaccharide (LPS) challenged condition. Three studies were conducted to find the effect of HESM supplement on post hatch chickens. In the first study, the chickens were fed either a control diet or diets containing 0.5% whey protein or HESM as supplement and evaluated at 5 weeks of age using growth, hematology, clinical chemistry, plasma immunoglobulins, and corticosterone as variables. The second and third studies were done to compare the effects of LPS on control and HESM fed birds at 5 weeks of age following at 4 and 24 h of treatment where the HESM was also sterilized with ethanol to deplete bacterial factors. HESM supplement caused weight gain in 2 experiments and decreased blood corticosterone concentrations. While LPS caused a significant loss in body weight at 24 h following its administration, the HESM supplemented birds showed significantly less body weight loss compared with the control fed birds. The WBC, heterophil/lymphocyte ratio, and the levels of IgG were low in chickens fed diets with HESM supplement compared with control diet group. LPS challenge increased the expression of pro-inflammatory cytokine gene IL-6 but the HESM fed birds showed its effect curtailed, also, which also, favored the up-regulation of anti-inflammatory genes compared with control diet fed chickens. Post hatch supplementation of HESM appears to improve performance, modulate immunity, and increase resistance of chickens to endotoxin.

## Introduction

Eggshells constitute a significant part of hatchery waste that consist of calcareous crust, shell membranes, proteins and peptides of embryonic origins, and many entrapped contaminants including microbes [1, 2]. Proteomic analysis of the eggshell membranes (ESM) showed the presence of over 200 proteins and peptides belonging to structural, antimicrobial, and cell-regulatory genre [3–5] with the hatchery eggshell membranes (HESM) further enriched with many blood derived proteins (Makkar et al., in preparation). We hypothesized that HESM as a feed supplement may be beneficial for post hatch poultry in the paradigm of mammalian milk which contain many similar proteins and peptides such as lactoferrin, lysozyme, albumin, and other factors that help gastrointestinal maturation and the development of immunity of neonates [6, 7]. However, the functional stability of these proteins to harsh processes such as drying, decontamination, and passage through the gastrointestinal tract is not known. Reports in the literature have shown the biological effects of different enzymes, antibodies, recombinant cytokines, and other bioactive protein additives in animal feed [8–13]. Previously, we showed that nutritional supplement of eggshell membrane (ESM) from fresh unfertilized eggs, given to the chickens during first 2 weeks post hatch, improved growth, increased serum immunoglobulins, and reduced several stress variables such as plasma corticosterone, heterophils, and heterophil/lymphocyte ratios[14]. The growth supportive effects of fetal proteins have also been demonstrated in other experimental models [15, 16]. The muco-adhesive egg shell membranes not only contain many adjuvant-like proteins and peptides [17, 18] but also act as carriers of different microbial antigens aiding the development of resistance or tolerance to pathogens. Hence, the objective of this study was to explore the effect of HESM supplements on the performance of post hatch chickens and their response to endotoxin stress, evaluating their growth, mortality, hematology, clinical chemistry, immunity, and stress variables.

## Materials and Method

### Preparation of HESM and its sterilization

Empty eggshells collected from hatchery waste were dried at room temperature to separate membranes from the shells and pulverized to powders and flakes with an IKA mill (Cole Parmer). The protein nitrogen content of the membrane powders, before and after mixing with feed, were estimated by Duma’s nitrogen analyzer using duplicate samples [14]. Three feeding experiments were conducted, Study 1 utilized intact HESM while both Studies 2 and 3 utilized HESM flakes sterilized with ethanol to reduce bacterial and endotoxin contaminants. In studies with ethanol sterilization, the HESM flakes were treated with 3 volumes (w/v) of reagent grade ethanol enough, to wet the flakes and air dried in a chemical hood without decantation. The effect of this treatment was evaluated using bacterial colony count assays [19] and the production of nitrite by HTC macrophages by endotoxin and bacterial factors [20]. Briefly, duplicate samples of untreated and ethanol treated HESM powders were extracted with sterile saline at the concentrations of 100 mg/ mL at room temperature for 2 h and centrifuged at 21,000 g. Respective supernatants were serially diluted with saline and 100 μl of each sample were spread on agar plates in triplicate and incubated for 24 h at 37°C to evaluate for bacterial growth. The same extracts were also evaluated for endotoxin activities using nitrite production by the HTC chicken macrophages following 24 h of stimulation and compared with Salmonella LPS (1μg/ml) used as a positive control [20].

### Experimental Schedule

The animal study protocols were approved by the Institutional Animal Care and Use Committee (IACUC) of the University of Arkansas. Newly hatched Cobb 500 male chicks were raised on floor pens at an approximate density of 0.8 square feet /bird with 23:1 light: dark schedule and provided feed formulated per National Research Council [21] specification and *ad libitum* water. The HESM was added at 0.5% level to broiler starter diet based on previous experiments. In Study 1 the effects of crude HESM and a comparable level of whey protein powder were tested on the growth performance and other physiological parameters of 5-wk-old chickens as described later. In Studies 2 and 3, the HESM powder was ethanol sterilized and used as the feed supplement where the effects of *Salmonella typhimurium* lipopolysaccharide (LPS) was evaluated following 4 and 24 h of its administration. In all experiments the chickens were first fed the diets containing the supplements for 14 days post hatch then switched to their regular diets for the rest of the time until necropsy. The birds were monitored daily for mortality, welfare, and evaluated weekly for body weight (BW) and feed consumption. The BW of the birds were measured before LPS injection and prior to necropsy in Studies 2 and 3.

In Study 1, 72 one day-old chicks were divided into 3 groups each with 24 birds in two replicate pens. The three groups received diets as follows: 1) control feed with no supplement, 2) feed containing 0.5% whey protein powder as a secondary control to find whether the effect was due to protein supplement alone, and 3) feed containing 0.5% HESM. Prior to necropsy, 6 birds from each pen (12/group) bled by cardiac puncture, blood collected in EDTA containing Vacutainer tubes for hematology and rapid serum tubes (BD Falcon) for clinical chemistry assays, respectively [14]. The birds were killed by cervical dislocation.

In Studies 2 and 3 the growth performance of the birds were measured along with other physiological changes including the effects of *Salmonella typhimurium* LPS (cat # HC4060 Sigma-Aldrich, St. Louis, MO). In Study 2, fifty day-old chickens were allocated into 2 groups and given feed with and without 0.5% sterile HESM supplement for 2 weeks post hatch as described above, then switched to regular feed for rest of the times through 5 weeks of age. On day 34, 12 birds in each group were injected LPS at a concentration of 1 mg/kg BW in saline, intramuscularly in the thigh, while the rest received equal volumes of saline injection. The effect of LPS was monitored visually for 5 h following injections with the BW measured after 24 h of injection. Prior to necropsy all chickens (n = 12/ treatment) were bled for hematology and clinical chemistry assays. At necropsy, the weights of selective organs from all birds were recorded.

In Study 3, the effect of LPS on splenic expression of selective genes with known association with different immune function were determined. Chickens from both control and HESM groups received either saline or LPS injection as described earlier. Four h after the injection 6 chickens from each group were killed and the spleens placed in liquid nitrogen for RNA extraction with the rest killed after 24 h to record BW and organ weights, respectively.

### Necropsy

The liver, heart, spleen, and bursa weights from all birds were used to calculate the percentage relative to BW. In Study 2, a cm length of ileum below the pancreatic loop was excised from each of 6 control and HESM fed birds and fixed in Carnoy’s fluid for ~5 h, transferred to 70% alcohol then processed for histology. Six micron paraffin sections were stained with periodic acid Schiff (PAS) hematoxylin staining and examined for villus health, mucous secretion, and gross abnormality by visual observation. The sections were photographed in BX Olympus microscope.

### Hematology

Blood cell counts along with hemoglobin content, mean corpuscular volume (MCV), hematocrit, microhematocrit (MCH), red blood cell distribution width (RDW), values were measured using EDTA anticoagulated blood using Cell-Dyn 3500 blood analysis system (Abbott Diagnostics, Abbott Park, IL) standardized for avian blood.

### Serum assays

The serum metabolic parameters were assayed using a clinical chemistry analyzer (Ciba Corning Diagnostics Corp, Medfield, MA). Corticosterone concentrations were measured using Detect X enzyme immunoassay kit^™^ (Arbor Assays, Ann Arbor, MI) and predetermined dilutions of sera [14]. The IgM, IgG, and IgA concentrations were similarly, determined using respective assay kits from Bethyl Laboratory (Montgomery, TX) with the serum diluted to 1:1000 with the manufacturer supplied buffer for IgA, 1:50,000 for IgG, and 1:20,000 for IgM, respectively. The concentrations of antibodies in the sera were calculated from their respective standard curves.

### Gene expression

The expressions of inflammation regulatory genes, pro-inflammatory (IL-1β, IL-6, IFN-γ), anti-inflammatory (IL-4, IL-10, IL-12), and immunosuppressive, wound repair supportive factors (TGF-β3 and vascular endothelial growth factor (VEGF) [22–24] were determined using splenic tissue RNA extract from respective treatments, and quantitative RT-PCR. Six frozen spleens from each treatment group were split into 4 quarters and ~ 100 mg of tissues from equivalent region of each spleen were extracted with Tri Reagent (Sigma-Aldrich) to prepare RNA. Complementary DNA (cDNA) was synthesized from RNA using qScript^™^ cDNA SuperMix (Quanta Biosciences) following manufacturer's suggested protocol. Quantitative real-time PCR was performed using SYBR^®^ green PCR master mix (Life technologies) in an ABI 7500 Real-Time PCR system (Applied Biosystems, Carlsbad, CA). A 25 μl reaction containing 5 μl cDNA (1 μg of RNA equivalent) and primers specific against chicken IL-1β, IL-4, IL-6, IL-10, IFN-γ, TGF-β3, VEGF and IL-12 (S1 Table), were subjected to PCR with an initial denaturation at 95°C for 10 minutes followed by 40 PCR cycles as follows: 95°C for 15 s and 58°C for 1 min. The expressions of target genes were analyzed by the 2^−ΔΔCt^ method [25] with 18S RNA as reference.

### Statistical analyses

The comparative results were evaluated using 2way Student’s t test and Duncan's multiple range tests using SAS software [26] and a *P*-value of ≤0.05 considered to be significant. The results are shown as mean ±SEM.

## Results

### HESM

**Fig 1:**
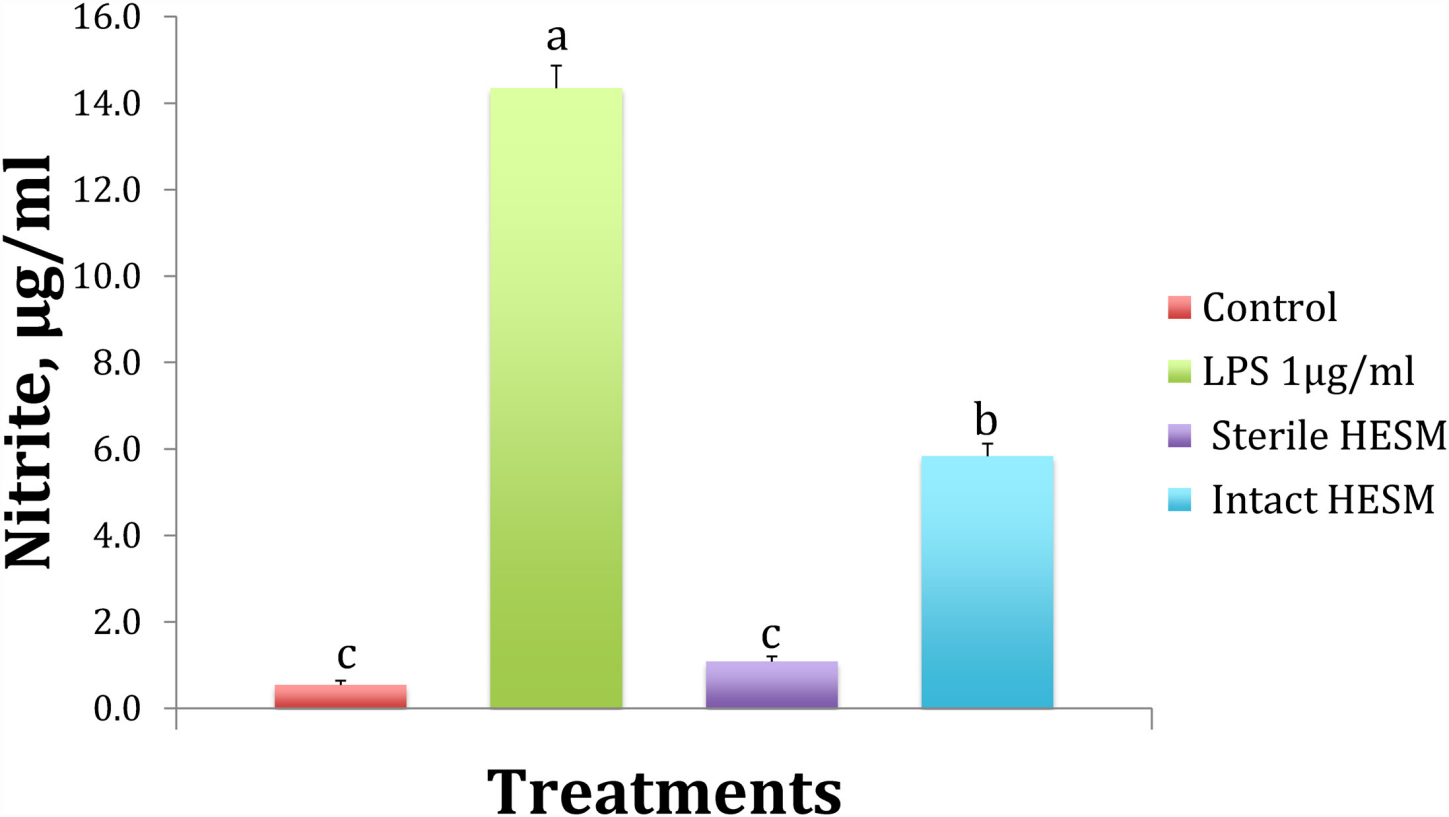
Nitrite production at 24 h by the HTC chicken macrophage in response to treatments with LPS and HESM extracts. Bars with different alphabets are statistically different (P ≤ 0.05).

The average protein nitrogen content of HESM was determined to be approximately 88% (w/w), the addition of which did not significantly alter the protein content of the feed (Control: 25.1% and HESM: 25.3%, n = 2 samples/group). The number of bacterial colonies showed a significant reduction from 30,000 / ml in untreated HESM extract to less than 5 colonies in ethanol treated HESM extract. Ethanol treatment also, reduced the endotoxin content of HESM, judged by a significantly low level of nitrite production by the HTC cells (Fig 1).

### Effect on BW and mortality

**Fig 2:**
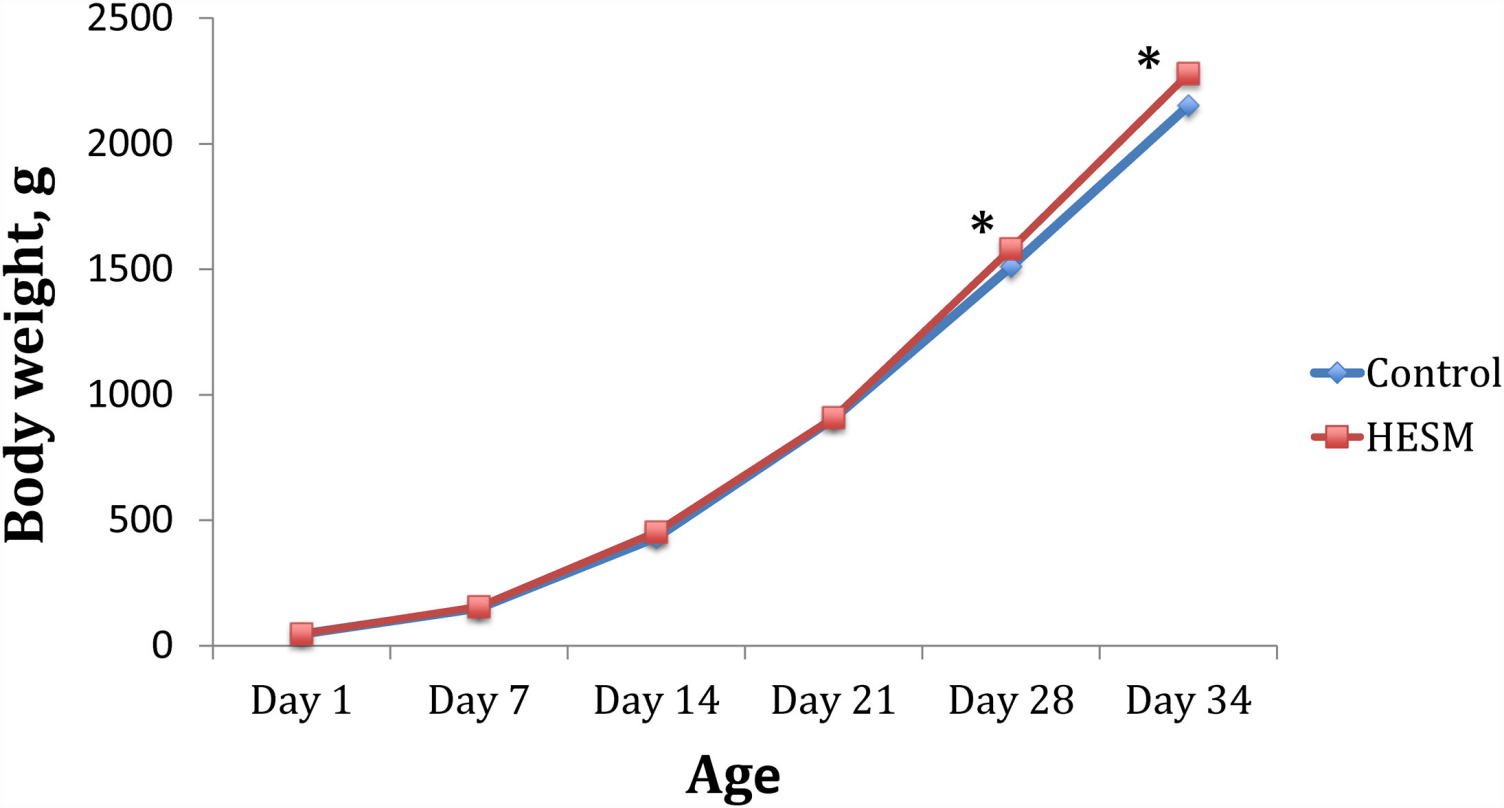
Weekly growth curve of chickens from day1 through 34 on diets with or without HESM supplement fed for first 2 weeks post hatch (n = 72–78).

In Study 1, there were no significant differences in body weight (BW) or relative organ weights of the birds given HESM supplement diet compared with either control or whey protein supplement groups (S2 Table). The body weight data combined from both Studies 2 and 3, showed significant increases in birds fed HESM supplement diet measured on both days 28 and 34 prior to their split for LPS treatment (Fig 2). The differences were however, not statistically discernible albeit, the birds in HESM group receiving saline had BW numerically higher on day 35 (Table 1). The cumulative mortality rate in all 3 experiments, combined, showed no significant differences between control and HESM fed chicks.

**Table 1:** Body weight (BW) and relative organ weights (% BW) of 35 days-old chickens fed control or sterile HESM supplement diet and treated with saline or LPS for 24 h. The results are combined from Studies 2 and 3 (n = 32–36).

	Saline		LPS	
Parameters	Control	HESM	Control	HESM
**BW (g)**	2238.27±33.38^a^	2327.23±40.77^a^	2021.30±37.02^c^	2135.38±28.55^b^
**Heart**	0.52±0.01^a^	0.51±0.01^a^	0.52±0.01^a^	0.52±0.01^a^
**Liver**	2.63±0.04^b^	2.48±0.06^b^	3.41±0.08^a^	3.30±0.09^a^
**Spleen**	0.12±0.01^a^	0.12±0.00^a^	0.13±0.01^a^	0.13±0.01^a^
**Bursa**	0.16±0.02^a^	0.13±0.01^b^	0.17±0.01^a^	0.13±0.01^b^
Mortality (%)*	9±1.53^a^	14.33^a^	-	-

Values with different superscripts in a row are significantly different (P≤0.05).

*Results are based on all 3 Studies.

### LPS effect

LPS treated chickens showed symptoms of sickness indicated by lack of activity, eyelid closure, and feed avoidance within 3 h of treatment, and decreases in BW at 24 h. The relative liver weights were significantly increased and bursa weights decreased in LPS treated chickens although there were no changes in heart and spleen weights. The chickens that received HESM showed lesser (p≤ 0.05) BW loss compared to control fed birds (Table 1).

### Hematology and serum chemistry

The results of the Study 2 and the effect of LPS is shown in Table 2. HESM treatment *per se* had no effect on lymphocyte (L), monocyte (M), heterophil (H), or basophil (B) percentages, and H/L ratios. On LPS treatment, there was an increase in the percentage of heterophils, monocytes, and basophils, and a reduction in lymphocyte counts in both groups tending to increase H/L ratios. The relative decrease in heterophil and increase in the lymphocyte counts in HESM fed birds, challenged with LPS, resulted in a significant decrease in H/L ratios compared with similarly treated control diet fed chickens (Table 2). There were minor other changes including increased hematocrit of HESM birds treated with LPS. HESM also produced a moderate decrease in serum protein, calcium, and magnesium concentrations some of which increased upon LPS treatment. LPS caused a decrease in serum iron and increase in triglyceride concentrations in both feed groups (Table 3). The cholesterol and HDL levels were down regulated in serum of control birds compared with HESM supplement fed birds and challenged with LPS. Neither alanine nor aspartate amino transferase levels were affected by HESM indicating the lack of liver toxicity.

**Table 2:** Hematology profiles of chickens fed control or diets containing HESM supplement and treated with LPS, Study 2 (n = 12).

	Saline		LPS	
Parameters	Control	HESM	Control	HESM
**WBC (10**^**3**^**/μL)**	49.76±1.42^a^	46.01±1.75^b^	54.09± 3.12^a^	45.60±3.0^b^
**Heterophil (H) (%)**	11.44±0.36^c^	12.62±0.46^c^	30.85±2.35^a^	22.94±3.14^b^
**Lymphocyte (L) (%)**	83.81±0.65^a^	82.31±0.70^a^	61.80±2.22^c^	69.92±3.10^b^
**Heterophil/ Lymphocyte (H/L)**	0.14±0.01^c^	0.15±0.01^c^	0.50±0.06^a^	0.32±0.05^b^
**Monocyte (M) (%)**	2.32±0.18^b^	2.75±0.28^b^	4.31±0.33^a^	4.01±0.26^a^
**Eosinophil (E) (%)**	0.02±0.01^a^	0.01±0.00^a^	0.02±0.00^a^	0.02±0.00^a^
**Basophil (B) (%)**	1.99±0.17^b^	2.3±0.18^b^	2.99±0.19^a^	3.03±0.17^a^
**Red blood cell (× 10**^**6**^**/μL)**	2.18±0.04^a^	2.18±0.04^a^	2.24±0.03^a^	2.33±0.02^a^
**Hemoglobin (g/dL)**	6.77±0.09^b^	6.80±0.085^b^	6.92±0.062^b^	7.17±0.073^a^
**Hematocrit (%)**	59.26 ±1.15^b^	60.27±0.98^b^	60.11±0.68^b^	63.02±0.65^a^
**Mean corpuscular volume (MCV)(fL)**	271.23±1.73^b^	276.16±1.69^a^	267.32±1.58^b^	271.07±1.16^b^
**Thrombocyte (k/μL)**	0.03±0.03^a^	0.00±0.00^a^	0.64±0.44^a^	0.003±0.00^a^

Values with different superscripts in a row are significantly different (P ≤ 0.05).

**Table 3:** Serum clinical chemistry variables of 5 week-old chickens fed with regular diet or the diet supplemented with 0.5% HESM, and challenged with LPS, Study 2, (n = 12).

	Saline		LPS	
Parameters	Control	HESM	Control	HESM
**Albumin (g/dL)**	1.15± 0.02^a,b^	1.08± 0.02^b^	1.17± 0.02^a^	1.19± 0.03^a^
**Glucose(mg/dL)**	213.15±4.18^a^	216.15± 3.64^a^	199.08± 4.14^b^	212.85± 5.63^a^
**Inorganic phosphate (mg/dL)**	3.31±0.16^b^	3.28± 0.13^b^	3.28± 0.17^b^	4.01± 0.21^a^
**Total protein(g/dL)**	3.12± 0.06^a^	2.76± 0.04^b^	3.15 ± 0.05^a^	3.22± 0.01^a^
**Alkaline phosphate (U/L)**	193.77± 24.24^a,b^	226.31± 28.60^a^	139.77 ± 14.90^b^	192.85±24.93^a,b^
**Alanine aminotransferase (U/L)**	3.85 ± 0.62^a^	2.49± 0.49^a^	2.75± 0.44^a^	3.85± 0.56^a^
**Aspartate Aminotransferase (U/L)**	309.45±17.4^a^	348.67±29.45^a^	324.95±18.49^a^	380.83±24.72^a^
**Blood urea nitrogen (mg/μL)**	1.61± 0.13^a^	1.05± 0.15^a^	1.33± 0.08^a^	1.30± 0.08^a^
**Magnesium (mEq/L)**	1.90± 0.05^a^	1.58± 0.04^b^	1.73± 0.05^a, b^	1.68± 0.11^b^
**Calcium (mg/dL)**	10.53± 0.23^a^	7.99± 0.26^c^	10.28± 0.29^a^	9.40± 0.27^b^
**Cholesterol (mg/dL)**	165.15± 6.91^a^	166.00± 4.16^a^	138.54±4.05^b^	161.31 ± 6.20^a^
**Creatinine kinase(U/L)**	546.8±83.43^a,b^	821.9 ± 166.59^a^	288.9± 36.06^b^	388.7± 46.39^b^
**Triglycerides (mg/dL)**	55.00± 4.86^b^	51.07± 3.28^b^	93.00± 5.49^a^	86.31± 6.93^a^
**High density lipoprotein (mg/dL)**	45.15± 2.81^a^	44.77± 1.28^a^	35.69± 1.24^b^	41.23± 1.61^a^
**Iron (μg/dL)**	99.54± 4.94^a^	93.85± 4.80^a^	55.46± 5.18^b^	57.77± 9.27^b^

Values with different superscripts in a row are significantly different (P ≤ 0.05).

### Serum immunoglobulins and corticosterone

In Study 1 there were no changes in serum IgM levels of chickens fed either whey protein or intact HESM supplement but the IgG levels showed decrease with HESM (S3 Table). Similar trend was observed in the 2^nd^ study which upon LPS treatment increased the serum IgM levels while the IgG remained unchanged in HESM fed birds. Neither treatment had any effect on serum IgA (Table 4). The serum corticosterone concentrations were consistently lower in both Studies 1 and 2 in HESM fed birds which with LPS treatment, increased moderately reaching to the same levels as in saline treated control birds (Table 4).

**Table 4:** Serum IgG, IgM, IgA, and corticosterone levels of chickens fed regular NRC diet or diets supplemented with ethanol sterilized HESM and challenged with LPS for 24 h, Study 2 (n = 12).

	Saline		LPS	
Parameters	Control	HESM	Control	HESM
**IgM (mg/ml)**	2.84±0.30^b.a^	1.93±0.24^b^	3.83±0.40^a^	3.50±0.47^a^
**IgG (mg/ml)**	4.78±0.68^a^	1.23±0.17^b^	3.53±0.53^a^	0.98±0.17^b^
**IgA (mg/ml)**	0.56±0.15^a^	0.54±0.22^a^	1.18±0.17^a^	0.81±0.39^a^
**Corticosterone (ng/mL)**	7.74±0.95^a^	5.01±0.53^b^	6.7±0.60^a,b^	6.58± 0.96^a,b^

Values with different superscripts in a row are significantly different (P ≤ 0.05).

### Gene expression

**Fig 3:**
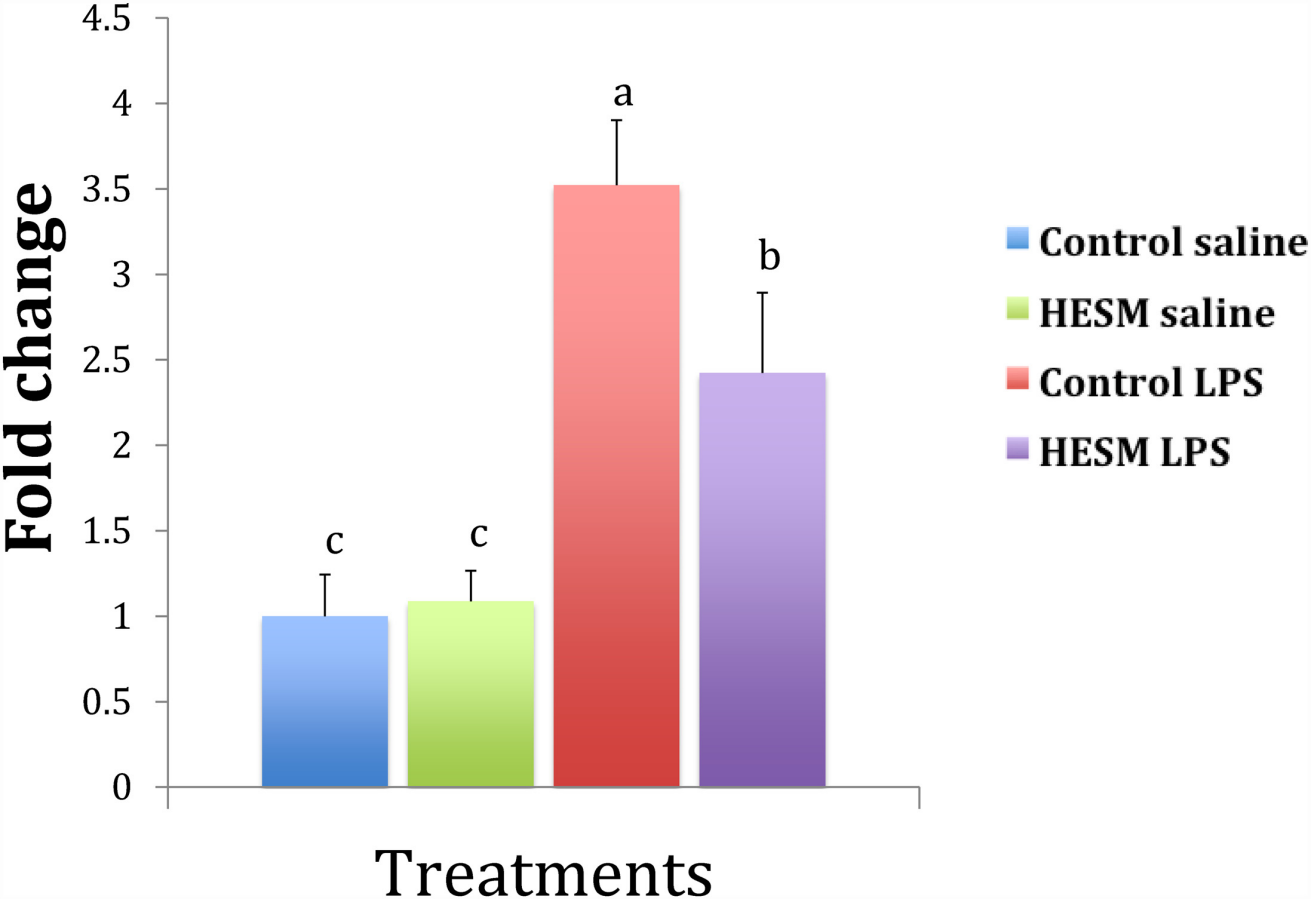
Splenic IL-6 gene expression in chickens fed control or HESM supplement diet and challenged with LPS or saline for 4 h (n = 6). Bars with different alphabets are significantly different (p≤0.05).

**Fig 4:**
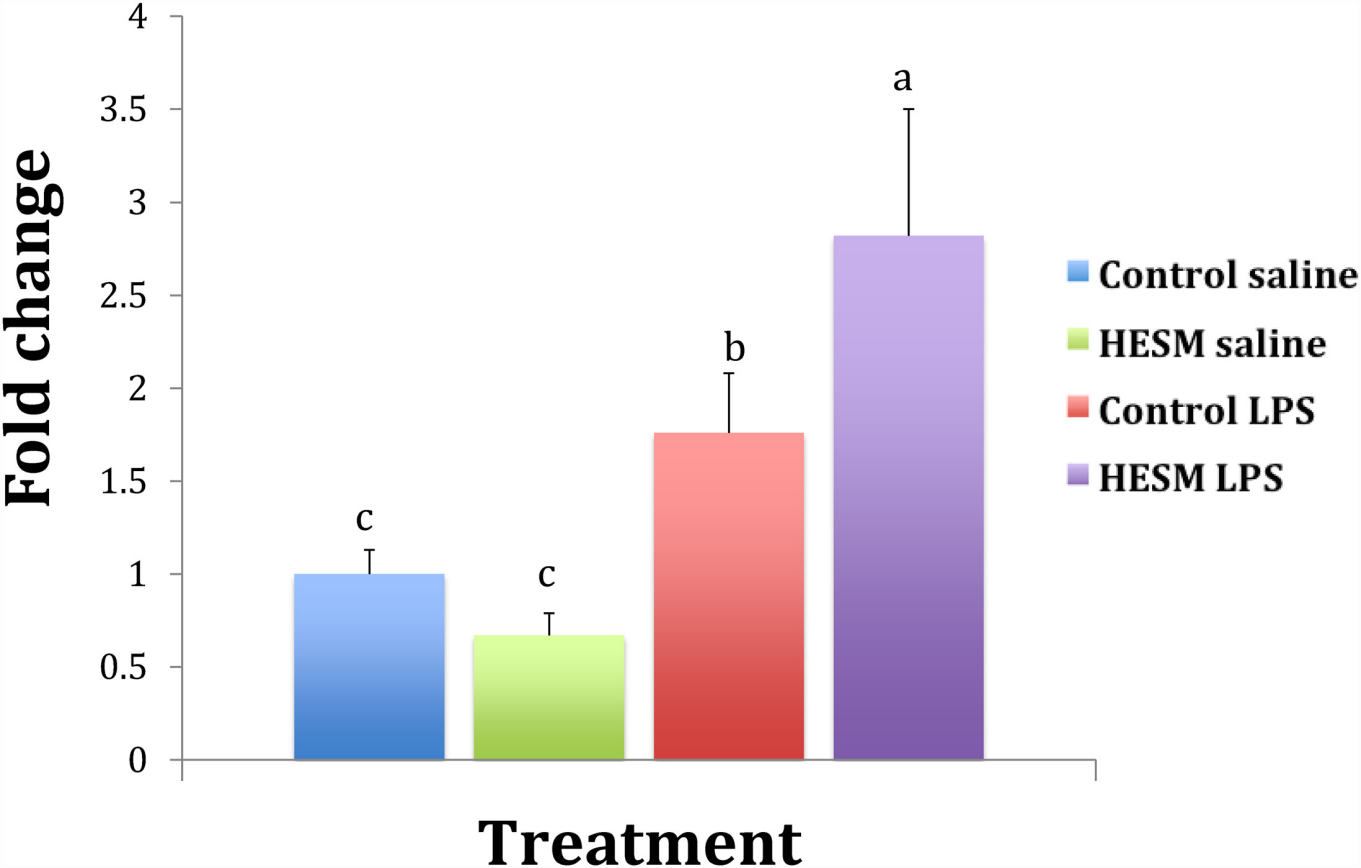
Splenic IL-10 gene expression of chickens fed control or HESM supplement diet and treated with LPS or saline for 4 h (n = 6). Bars with different alphabets are statistically different (P ≤ 0.05).

**Fig 5:**
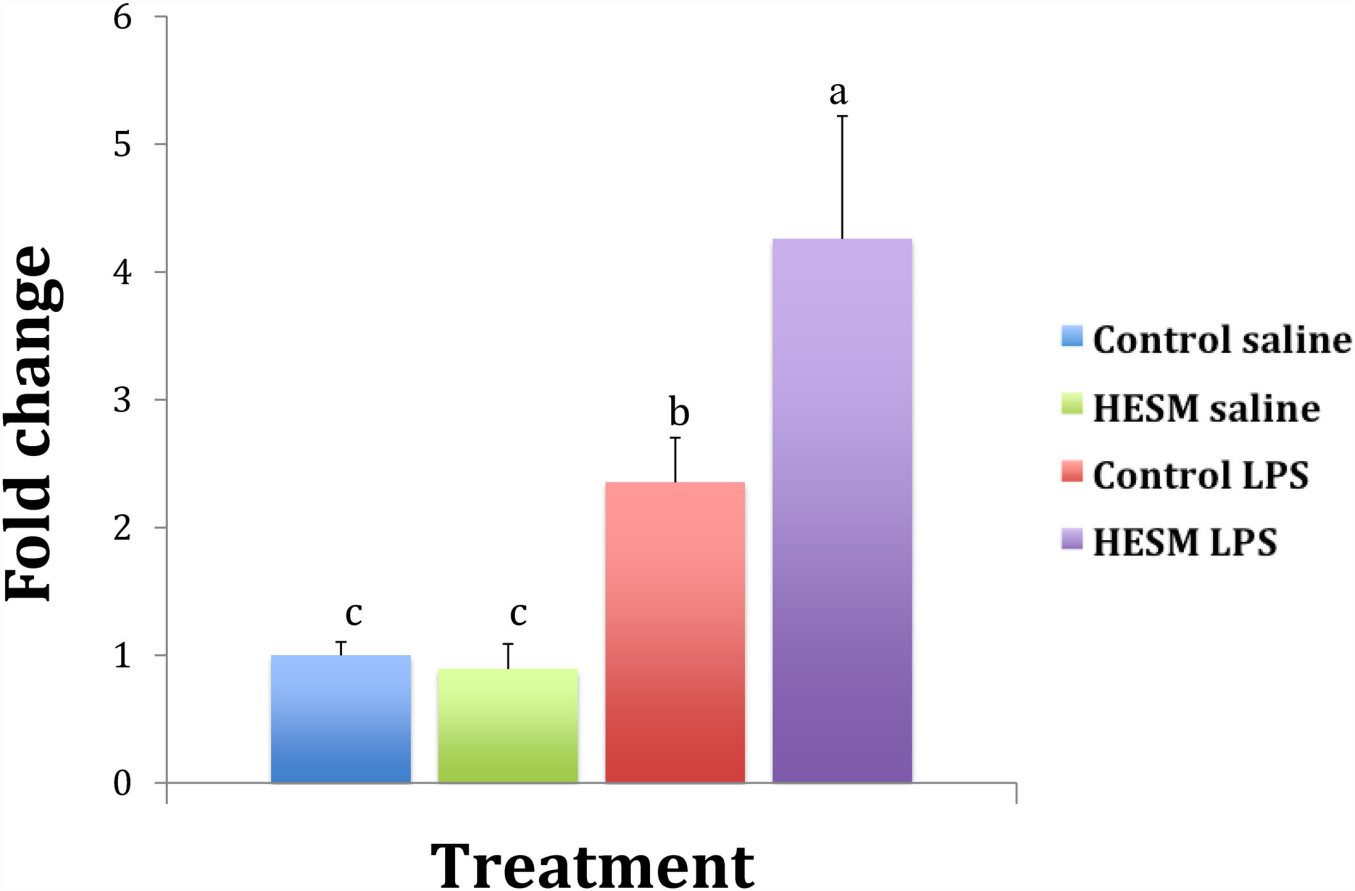
Comparison of splenic IL-4 gene expression in chickens fed control or HESM supplement diet and challenged with LPS or saline for 4 h (n = 6). Bars with different alphabets are statistically different (P ≤ 0.05).

The splenic gene expression results are shown in Table 5. Chickens fed regular diet and challenged with LPS had a significant increase in IL-6 gene expression compared with HESM fed birds (Fig 3). The anti-inflammatory gene IL-10 showed a significant increase in the HESM group challenged with LPS compared with control fed chickens challenged with LPS (Fig 4). The IL-4 gene was downregulated in HESM birds but on LPS treatment its expression was significantly higher compared with controls (Fig 5). There were no change in the expressions of IFN-ᵧ or IL-12. The TGF-β expression showed a significant decrease by LPS treatment in both control and HESM supplement fed groups whereas the VEGF was downregulated in HESM birds regardless of LPS treatment.

**Table 5:** Study 3. The expression of splenic genes quantified by RT-PCR in birds fed with or without HESM and treated with LPS or saline for 4 h (n = 6).

	Saline		LPS	
Genes	Control	HESM	Control	HESM
**IL-1**	1.00± 0.24^b,a^	0.75±0.11^b^	1.32±0.25^b,a^	1.83±0.61^a^
**IL-6**	1.00±0.24^b^	1.08±0.17^b^	3.52±0.37^a^	2.42±0.49^b^
**IL-10**	1.00± 0.10^b,c^	0.89±0.19^c^	2.3±0.34^b^	4.26±0.95^a^
**IFN-γ**	1.00±0.28^a^	0.99±0.26^a^	0.68±0.13^a^	1.15±0.31^a^
**TGF-β**	1.00±0.20^a^	0.97±0.14^a^	0.28±0.04^b^	0.30±0.04^b^
**IL-12**	1.00±0.13^a^	1.43±0.32^a^	1.13±0.26^a^	1.64±0.19^a^
**VEGF**	1.00±0.13^a^	0.32±0.08^b^	0.86±0.11^a^	0.31±0.04^b^
**IL-4**	1.00±0.13^b,c^	0.67±0.12^c^	1.76±0.32^b^	2.82±0.68^a^

Values with different superscripts in a row are significantly different (P≤0.05).

### Histology

There were no differences in the overall health of intestine between the control and HESM diet fed birds judged by villus morphology, muscularis, and mucus deposition (S1 Fig).

## Discussion

Our results show that feeding HESM can be beneficial to chickens particularly decreasing stress and improving resistance to LPS-induced changes. These results are consistent with our previous report where the egg shell membranes (ESM) from fresh unfertilized eggs improved the performance of 3 week old chickens with respect to body weight, downregulated corticosterone, and other stress parameters[14]. In the previous study with fresh ESM as supplement we observed an increase in the serum levels of both IgG and IgM at 3 weeks post feeding but in the present study the IgM levels appeared not to be affected that can be due to later sampling time of 5 weeks when the early response to antigens may have subsided [27–29]. However, the cause of IgG downregulation in HESM treated birds is not understood.

Weight loss is a hallmark of endotoxemia in both mammals and birds which is mediated through several pro inflammatory cytokines such as IL-1, IL-6, and TNF-α [30–33]. These cytokines not only cause hypophagia but also promote protein catabolism leading to BW loss [34]. The HESM appears to curb the effect of endotoxin induced weight loss also, modify the expression of splenic cytokine genes that are associated with inflammation [35–39]. There was not only the downregulation of serum corticosterone but also another stress marker heterophil to lymphocyte ratio [40] in HESM fed birds that could account for their improved performance and resistance to endotoxin. Glucocorticoids are not only anti-anabolic but also immunosuppressive [41]. Lower stress can improve feeding and decrease susceptibility to pathogens in poultry [42]. However, the mechanism by which the ESM lower the stress parameters is not understood since the effect appears to persist beyond discontinuation of feeding HESM. Hypothetically a decreased serum level of adrenal steroids can be expected upon endocrine exhaustion under conditions such as chronic endotoxemia. However, the HESM fed chickens showed no sign of sickness judged from their BW, intestinal pathology, and blood profiles.

Although the expression of IL-6 gene was elevated with LPS treatment in both groups, it was significantly lower in birds with HESM. Similarly, the expressions of IL-4 and IL-10 genes in the spleen were upregulated both of which are considered as anti-inflammatory cytokines implicated in the development of immune tolerance [43, 44]. Anti-inflammatory effect of natural ESM has been reported in experimental models of joint inflammation where the effect was attributed to the proteoglycan content of the preparation [45, 46]. Similar results were also reported by Shi et.al in mice that showed the hydrolysate of eggshell membrane providing protection against dextran sodium sulfate induced intestinal inflammation [47]. The TGF-β expression was lower in both feed groups injected with LPS while the VEGF showed consistently lower expression in HESM birds. Since these growth factors help tissue repair and angiogenesis associated with the resolution of inflammation, [23] their downregulation during early phases of inflammation is likely. However, the decrease in VEGF expression in birds fed HESM treatment is not understood. Whether the patterns of expressions of pro and anti-inflammatory cytokines have any relevance to curb the LPS induced body weight loss in HESM fed chickens is not known. Evidently, a modified immune response due to HESM confers resistance to endotoxin induced changes. As the susceptibility to infection can increase in immunocompromised individual likewise, it could confer tolerance to disease in immune strengthened birds.

There were no significant differences in serum IgM or IgA levels of chickens fed either control or HESM diet with or without LPS challenge. By contrast, the IgG levels were reduced in birds fed HESM that did not substantially change upon LPS treatment. Hypogammaglobinemia with normal IgM and IgA have been noted in human patients with physical trauma such as burn and nephrosis [48]. But the chickens with HESM diet had neither physical trauma nor their clinical chemistry showed any indication of dysregulated kidney function such as hypoalbumenemia and hyperlipidemia that can be associated with nephrotic conditions. The HESM induced downregulation of serum corticosterone is consistent with our previous results with ESM [14]. We presume that post hatch exposure to HESM which is laden with different regulatory proteins and peptides and the remnants of bacterial and parasite contaminants could likely condition the neuro-immune system lowering the disposition of birds to stress and better tolerance to LPS. In newly hatched birds, as in mammalian neonates, the immune and neuroendocrine system is immature and prone to epigenetic conditioning. At this stage not only the maternal but also other biodiverse factors such as diets, and microbes likely could provide signals that can shape immunity and establish both tolerance and resistance to pathogens [49–52]. There is increasing evidence showing the neonatal exposure to stress, diets and microbiome bringing upon long term effect on immunity, health, and wellbeing of individuals [53, 54]. Besides, the enteric system has the second largest density of neurons that can be impacted by bioactive factors to influence immunity. It is now known that the immune functions of lymphoid organs such as spleen is prone to control through neural output of autonomic system and T cell regulation subject to cholinergic output [55, 56]. Thus, the bioactive embryonic factors in HESM modulating the immune response of chickens remains a possibility. Also, it is well recognized that maternal factors such as milk along with exposure to microbiome are important factors for establishing disease resistance and post-natal conditioning in mammals [57]. The numerous regulatory proteins and peptides present in the ESM thus, could simulate those effects in chickens.

From the foregoing discussion it is evident that post hatch feeding HESM supplement is beneficial to chickens because it can improve their wellbeing and resistance to harmful effects of LPS. Whether the effects are due to the bioactive proteins and peptides or other factors is not known. Very little is known as to whether and how food associated bioactive proteins influence immunity because many omnivorous birds and mammals rely on fresh protein sources for their early nutrition which may provide epigenetic conditioning to the immune system and build their resistance against common contagions. The postnatal immune system being immature but plastic, certainly provides opportunity for nutritional modulation to build better immunity [58]. In conclusion, our results show that HESM supplement can be a sustainable feed additive to improve immunity and health physiology of poultry.

## Supporting Information

S1 Fig Histology of intestine sections of control and HESM fed birds (magnification X400). (DOCX)

S1 Table PCR primers and accession numbers of target genes for chicken cytokines and growth factors. (DOCX)

S2 Table Study 1, body weight (BW) and the relative organ weights (% BW) of 5 week-old chicken fed control and diets with 0.5% whey protein powder or 0.5% HESM supplement (n = 20–23). (DOCX)

S3 Table Study 1, serum IgG, IgM, and corticosterone levels of chickens fed with regular NRC diet and 0.5% whey protein or HESM supplement diet. The results are shown as mean ± SEM. (n = 12/ group).
(DOCX)
