# Supplementary figures and images for: Nutritional Supplement of Hatchery Eggshell Membrane Improves Poultry Performance and Provides Resistance against Endotoxin Stress
Source: PLoS One. 2016 Jul 27;11(7):e0159433. doi: 10.1371/journal.pone.0159433 (PMC4963089; doi:10.1371/journal.pone.0159433)

**Figure S1**

.


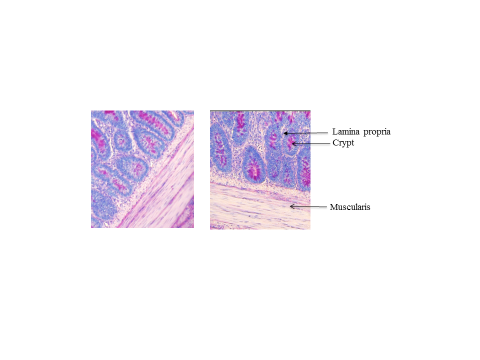


Control HESM

Supplement: S1 Fig — (DOCX) [file pone.0159433.s001.docx]
