# Supplementary material for: Nutritional Supplement of Hatchery Eggshell Membrane Improves Poultry Performance and Provides Resistance against Endotoxin Stress
Source: PLoS One. 2016 Jul 27;11(7):e0159433. doi: 10.1371/journal.pone.0159433 (PMC4963089; doi:10.1371/journal.pone.0159433)
Supplement: S1 Table — (DOCX) [file pone.0159433.s002.docx]

**Table S1.**

| **Target genes** | **Accession number** | **Primer sequences** | **Length of product (bp)** |
| --- | --- | --- | --- |
| **IL-1β** | NM_204524.1 | SF：CGAGGAGCAGGGACTTTGC  SR：GAAGGTGACGGGCTCAAAAA | 71 |
| **IL-6** | NM_204628.1 | SF：GCTTCGACGAGGAGAAATGC  SR:GGTAGGTCTGAAAGGCGAACAG | 63 |
| **IL-10** | NM_001004414.2 | SF：CGCTGTCACCGCTTCTTCA  SR：CGTCTCCTTGATCTGCTTGATG | 63 |
| **IFN-γ** | NM_205149 | SF：AAAGCCGCACATCAAACACA  SR：GCCATCAGGAAGGTTGTTTTTC | 64 |
| **TGF-β3** | NM_205454.1 | SF：TGCGGCCAGATGAGCAT  SR：TGCACATTCCTGCCACTGA | 55 |
| **18S rRNA** | NC_006088.3 | SF：TCCCCTCCCGTTACTTGGAT  SR：GCGCTCGTCGGCATGTA | 60 |
| **IL-12** | NC-46430425 | SF:TGCCCAGTGCCAGAAGGA  SR:TCAGTCGGCTGGTGCTCTT | 57 |
| **VEGF-A** | GI 160358852 | SF:AAATTCACAGACTCACGTTGCAA  SR: ATCTGCAAGTGCGCTCGTTT | 61 |
| **IL-4** | NM_0010079.1 | SF: GCTCTCAGTGCCGCTGATG  SR: GAAACCTCTCCCTGGATGTCAT | 60 |
