# Supplementary material for: Nutritional Supplement of Hatchery Eggshell Membrane Improves Poultry Performance and Provides Resistance against Endotoxin Stress
Source: PLoS One. 2016 Jul 27;11(7):e0159433. doi: 10.1371/journal.pone.0159433 (PMC4963089; doi:10.1371/journal.pone.0159433)
Supplement: S2 Table — (DOCX) [file pone.0159433.s003.docx]

**Table S2.**

| **Parameters** | **Control** | **0.5% whey protein** | **0.5% HESM** |
| --- | --- | --- | --- |
| **BW (g)** | 2153.95±45.97^a^   \|  \|  \| \| --- \| --- \| | 2219.65±37.20^a^ | 2148.00±37.74^a^ |
| **Heart** | 0.56±0.02^a^ | 0.53±0.02^a^ | 0.57±0.02^a^ |
| **Liver** | 2.20±0.04^a^ | 2.18±0.05^a^ | 2.3±0.06^a^ |
| **Spleen** | 0.11±0.01^a^ | 0.12±0.01^a^ | 0.11±0.01^a^ |
| **Bursa** | 0.15±0.01 ^a^   \|  \| \| --- \|  \|  \| \| --- \|  \|  \|  \| \| --- \| --- \| | 0.16±0.02^a^ | 0.16±0.01^a^ |
|  |  |  |  |
|  |  |  |  |

Values with different superscripts in a row are significantly different (P<0.05).
