# Supplementary material for: Nutritional Supplement of Hatchery Eggshell Membrane Improves Poultry Performance and Provides Resistance against Endotoxin Stress
Source: PLoS One. 2016 Jul 27;11(7):e0159433. doi: 10.1371/journal.pone.0159433 (PMC4963089; doi:10.1371/journal.pone.0159433)
Supplement: S3 Table — The results are shown as mean ± SEM. (n = 12/ group). (DOCX) [file pone.0159433.s004.docx]

**Table S3.**

| **Parameters** | **Control** | **0.5% whey protein** | **0.5% HESM** |
| --- | --- | --- | --- |
| IgG (mg/mL) | 1.20±0.07^a^ | 1.07±0.06^a^ | 0.82±0.07^b^ |
| IgM (mg/mL) | 2.69±0.24^a^ | 2.54±0.30^a^ | 2.60±0.22^a^ |
| Corticosterone (ng/mL) | 5.75±0.77^a^ | 4.26±0.33^a,b^ | 3.81±0.41^b,c^ |

Values with different superscripts in a row are significantly different (P ≤ 0.05).
